# Spatio-Temporal COVID-19 Modeling: A Global Systematic Review of Data Integration, Equity, and Lessons for Pandemic Preparedness

**DOI:** 10.3390/ijerph23050627

**Published:** 2026-05-08

**Authors:** Petra Norlund, Jamal Jokar Arsanjani, Jesper M. Paasch

**Affiliations:** 1 Department of Sustainability and Planning, Aalborg University Copenhagen, 2450 Aalborg, Denmark; jja@plan.aau.dk; 2 Department of Computer and Geospatial Sciences, University of Gävle, 801 76 Gävle, Sweden; jesper.paasch@hig.se

**Keywords:** COVID-19, spatio-temporal modeling, spatial epidemiology, global health, FAIR data, health equity, pandemic preparedness

## Abstract

**Highlights:**

**Public health relevance—How does this work relate to a public health issue?**

- It synthesizes global evidence on spatio-temporal COVID-19 models to understand how infectious diseases spread across space and time.
- It examines how publicly available data and modeling approaches supported surveillance, forecasting, and intervention planning during the pandemic.

**Public health significance—Why is this work of significance to public health?**

- It identifies critical gaps in data integration, geographic coverage, and equity that affect the effectiveness of pandemic response systems.
- It demonstrates that incorporating spatial, temporal, and socio-demographic factors improves the accuracy and policy relevance of public health models.

**Public health implications—What are the key implications or messages for practitioners, policy makers and/or researchers in public health?**

- It highlights the need for FAIR-aligned, multi-source, and equity-aware data infrastructures to strengthen future pandemic preparedness.
- It emphasizes that spatio-temporal models are most effective for short-term decision support and should be integrated into GIS-based tools for targeted interventions.

**Abstract:**

The COVID-19 pandemic generated an unprecedented volume of spatially and temporally resolved data, enabling rapid development of spatio-temporal models for surveillance, forecasting, and policy support. However, the evolution, geographic distribution, and equity implications of these models remain insufficiently synthesized. This study presents a global systematic review of 363 peer-reviewed studies published between January 2020 and August 2025 using publicly available data. Following PRISMA 2020 guidelines, studies were classified by geographic scale, modeling approach, data streams, and analytical purpose. The results indicate that Bayesian and compartmental models remained dominant throughout the pandemic, although methodological diversity increased over time with the growing use of machine learning and hybrid frameworks integrating mobility, environmental, and socio-demographic data. Data integration was more common than previously reported. Approximately 30% of studies relied on a single data stream, while 70% incorporated multiple sources, although most multi-source approaches combined only two data types and relatively few studies integrated three or more. Geographic coverage was uneven, with a strong concentration of studies in high-income regions and persistent underrepresentation of low- and middle-income contexts. Models incorporating finer spatial scales and socio-demographic variables more frequently supported geographically targeted interpretation of risk, vulnerability, testing access, and intervention needs. Overall, the findings highlight the importance of multi-source data integration, improved geographic representativeness, and transparent uncertainty communication, alongside the need for FAIR-aligned and equity-aware data infrastructures to strengthen future pandemic preparedness.

## 1. Introduction

The COVID-19 pandemic demonstrated that infectious disease dynamics are inherently spatial and temporal. Outbreaks emerged unevenly across regions, spread through interconnected places, and evolved rapidly over time, placing localized pressure on healthcare systems. These dynamics made clear that effective public health responses require models capable of integrating where infections occur with how they change over time. Spatio-temporal modeling, which explicitly links geographic context with temporal dynamics, therefore became central to outbreak detection, forecasting, and the evaluation of interventions during the pandemic [1,2,3]. With five years of accumulated research now available, it is possible to assess how these models were developed, applied, and adapted across different phases of the pandemic.

Geographic context is central to understanding infectious disease dynamics because risk, exposure, and healthcare resources are spatially heterogeneous. Transmission processes operate across interconnected locations rather than within isolated administrative units, generating spatial dependence and cross-regional spillover. Spatial epidemiology provides statistical and computational frameworks for quantifying these dependencies and for modeling how transmission evolves jointly across space and time. Geographic Information Systems (GIS) and spatial analytic methods were widely used during the COVID-19 pandemic to visualize incidence patterns, identify hotspots, and support subnational surveillance [3,4]. However, descriptive mapping alone is insufficient for inference. Robust epidemic analysis requires models that explicitly incorporate spatial structures such as adjacency matrices, spatial random effects, or network connectivity—together with temporal dynamics. Integrating these components is essential for capturing transmission heterogeneity and informing targeted interventions.

As the pandemic unfolded, spatio-temporal models based on publicly available data were developed across a wide range of geographic contexts, highlighting the geographically uneven impacts of COVID-19. Using open data sources such as case counts, mortality records, mobility indicators, and demographic information, researchers applied predominantly in data-rich high-income countries, with more limited representation from regions with constrained surveillance capacity. While multiple data types were often available, their integration within individual models remained uneven, with many studies combining only a limited number of data streams. Models were frequently adapted to local conditions, reflecting differences in data availability, population structure, mobility patterns, and socioeconomic context. This global body of work demonstrates both the flexibility of spatio-temporal modeling and its dependence on underlying data infrastructures. The COVID-19 pandemic generated unprecedented volumes of spatially and temporally resolved data, prompting rapid methodological experimentation across epidemiology, geography, and data science. This led to the increasing diversification of modeling approaches, including the growing use of machine learning and hybrid models, although statistical and compartmental frameworks remained dominant throughout the pandemic [5]. In parallel, a smaller but growing body of research has developed hybrid modeling approaches that integrate machine learning with mechanistic, statistical, or physically grounded frameworks to improve interpretability and robustness. For example, Sakovich et al. and Briz-Redón [6,7] propose a neural operator that combines dynamic mode decomposition with deep learning to approximate spatio-temporal processes governed by partial differential equations, illustrating how hybrid approaches can bridge data-driven learning and physically informed modeling, thereby strengthening the methodological scope of the review. This diversity reflects both methodological innovation and the uneven capacity to integrate complex data sources across geographic contexts.

Despite the rapid proliferation of spatio-temporal COVID-19 studies, existing reviews have been limited in scope. Early syntheses focused primarily on the first pandemic wave or on specific regions [5,7], while others emphasized methodological families or applications. No prior study has provided a comprehensive global synthesis of spatio-temporal COVID-19 models built on publicly available data across the full pandemic period from 2020 to 2025. This gap is significant, as it limits understanding of how modeling strategies evolved over time, how open data were used across geographic contexts, and how methodological choices shaped policy-relevant insights.

Moreover, spatio-temporal COVID-19 modeling faced persistent challenges. Data availability and quality varied widely across regions, particularly in low- and middle-income countries, constraining geographic coverage and comparability [8,9]. Model transferability across contexts remained limited, and long-term forecasting proved difficult as viral evolution, behavioral change, and policy shifts introduced strong non-stationarity [1,10]. Although many studies relied on publicly available data, adherence to FAIR (Findable, Accessible, Interoperable, Reusable) principles was uneven, with studies generally being findable but often lacking accessibility, interoperability, and reusability.

Against this background, this study presents a global systematic review of spatio-temporal COVID-19 modeling studies based on publicly available data published between January 2020 and August 2025. The review has three objectives: (i) to characterize the diversity of spatio-temporal modeling approaches applied across geographic contexts, (ii) to document the types of open data used and their integration across models, and (iii) to synthesize key methodological, geographic, and policy-relevant lessons for future pandemic preparedness. By situating spatio-temporal modeling within a health geography framework and emphasizing open, FAIR-aligned data practices, this review provides a foundation for developing adaptive, geographically grounded, and equitable modeling approaches for future global health emergencies.

From a global health perspective, understanding how spatio-temporal models performed across heterogeneous data environments is critical for future pandemic preparedness. The uneven geographic distribution of modeling capacity and data availability identified in this review highlights structural inequities that shape global surveillance and response capabilities. Addressing these gaps is essential for building inclusive, scalable, and ethical global health intelligence systems. By synthesizing methodological, geographic, and data integration dimensions simultaneously, this review provides one of the first global assessments of how spatio-temporal COVID-19 modeling evolved across the full pandemic period.

## 2. Materials and Methods

### 2.1. Literature Search Strategy

To identify studies applying spatio-temporal models to COVID-19, we conducted a comprehensive literature search covering the period from January 2020 through August 2025. The core search string combined epidemiological, spatial, and modeling terms using Boolean operators: (‘COVID-19’ OR ‘SARS-CoV-2’) AND (‘spatio-temporal’ OR ‘spatiotemporal’ OR ‘spatial-temporal’) AND (‘model*’ OR ‘forecast*’ OR ‘prediction’) AND (‘spatial epidemiology’ OR ‘GIS’ OR ‘geographic*’). Additional terms related to open or publicly available data were applied during screening rather than as database filters.

Searches were conducted in Web of Science, Scopus, PubMed, Google Scholar, and arXiv between 12 January 2025 and 11 August 2025, with Google Scholar and arXiv used to complement indexed databases and capture emerging literature. Database-specific search strings were adapted as needed, and forward and backward citation tracking was used to identify additional relevant studies not retrieved through database queries.

This broad and iterative strategy was designed to capture the full range of spatio-temporal modeling approaches developed during the COVID-19 pandemic.

### 2.2. Eligibility Criteria

Studies were included if they met all the following criteria:

(i) Explicitly integrated both spatial and temporal components in modeling COVID-19 dynamics;
(ii) Relied primarily on publicly available or accessible data sources, such as confirmed cases, hospitalizations, mortality, mobility, vaccination, genomic, or environmental data;
(iii) Were published in English between January 2020 and August 2025;
(iv) Reported sufficient methodological detail to allow interpretation of the modeling framework.

Studies were excluded if they:

(i) Focused exclusively on spatial or temporal analysis without integrating both dimensions;
(ii) Relied solely on proprietary or inaccessible data;
(iii) Lacked methodological transparency;
(iv) Addressed purely clinical or genomic questions without a spatial component.

These criteria were chosen to ensure reproducibility, comparability, and alignment with principles of spatial epidemiology and open science [4,5].

### 2.3. Study Selection and Screening

**Figure 1:**
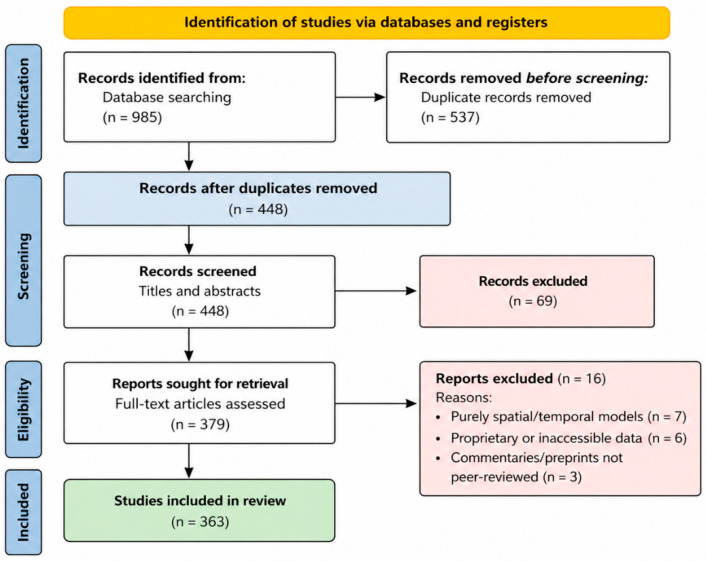
PRISMA 2020 flow chart illustrating the identification, screening, eligibility assessment, and inclusion of studies in the systematic review of quantitative spatio-temporal COVID-19 models based on publicly available data (adapted from [11]).

The initial search yielded 985 records, with an additional 55 identified through citation tracking. After removal of duplicates and screening of titles and abstracts, full texts were assessed for eligibility. A total of 363 studies met the inclusion criteria. This systematic review was conducted and reported in accordance with the PRISMA 2020 guidelines. A completed PRISMA checklist is provided in Supplementary Material File S1. The review protocol was not preregistered because the study aimed to synthesize rapidly evolving methodological literature rather than estimate pooled epidemiological effects. The study identification, screening, eligibility, and inclusion process is documented using a PRISMA flow diagram (Figure 1), following established systematic review guidelines.

#### Screening Procedure and Reviewer Roles

Study selection and data extraction were primarily conducted by a single reviewer due to the exploratory and methodological focus of the review. To enhance consistency and reduce potential bias, screening criteria were applied systematically across all records, and ambiguous cases were revisited iteratively during the screening process. While duplicate independent screening and extraction were not performed, a structured protocol based on PRISMA 2020 guidelines was followed, and decisions were documented to ensure transparency and reproducibility. Given the large and heterogeneous body of literature and the aim of providing a global methodological synthesis rather than estimating pooled effect sizes, this approach was considered appropriate. However, we acknowledge that the absence of duplicate screening may increase the risk of selection bias. This approach is consistent with prior systematic reviews of complex modeling literature where duplicate screening is not always feasible.

### 2.4. Data Extraction

For each included study, we extracted standardized information on:

- Geographic coverage and spatial scale;
- Temporal scope; primary modeling approach (e.g., Bayesian spatio-temporal models, SEIR/SIRD variants, machine learning, agent-based simulations);
- Data sources and types of open data used; and primary analytical purpose (e.g., forecasting, hotspot detection, intervention evaluation, surveillance). Attention was given to whether studies integrated multiple data streams within a single modeling framework or relied on a limited subset of available data.

All extracted information was compiled into a structured database to support systematic comparison across studies. To capture the extent to which studies supported practical decision-making, we additionally recorded whether model outputs were described as directly relevant for geographically targeted interpretation or intervention. This included, for example, identification of hotspots, small-area risk estimation, assessment of vulnerable populations, evaluation of intervention effects, or support for resource allocation. This characteristic was assessed qualitatively based on the authors’ stated objectives and reported outputs and was used descriptively in the synthesis rather than as a formal quantitative performance metric. Where reported, information on model validation and stated predictive limitations was extracted descriptively, including authors’ discussion of short-term versus longer-horizon forecasting and performance during periods of rapid epidemiological change. Because validation metrics, forecast horizons, and outcomes varied substantially across studies, no formal pooled quantitative comparison of predictive performance was undertaken. Additional references for studies included in the review corpus but not directly discussed in the main text are provided in Supplementary Material File S2. All extracted information was compiled into a structured database to support systematic comparison across studies. The full list of the 363 included studies and extracted study characteristics is available in Supplementary Material File S3.

### 2.5. Study Classification and Synthesis Framework

To enable synthesis across heterogeneous literature, studies were classified along five analytical dimensions:

I. Geographic scale (global, national, regional, local);
II. Modeling family (Bayesian/statistical, compartmental, machine learning, agent-based, mechanistic–statistical hybrid, or hybrid);
III. Data streams used (including single- and multi-source integration) (e.g., case incidence, mobility, mortality, vaccination, genomic, wastewater);
IV. Analytical purposes (forecasting, risk mapping, intervention assessment, early warning); and
V. Policy or decision-support relevance.

To ensure consistency in classification, modeling approaches were organized into a formalized taxonomy of spatio-temporal model families. This taxonomy was developed inductively from the reviewed literature and groups models into seven categories based on their underlying methodological paradigm: (i) Bayesian/statistical spatio-temporal models, (ii) compartmental models (e.g., SEIR/SIRD variants), (iii) machine learning and deep learning models, (iv) graph-based spatio-temporal models, (v) agent-based models, (vi) mechanistic–statistical hybrid models, and (vii) multi-source hybrid frameworks. Machine learning models were defined as primarily data-driven approaches without explicit epidemiological structure, whereas hybrid models explicitly combined data-driven methods with mechanistic, statistical, or physically based components.

Classification was based on the primary modeling logic described in each study. Where studies combined multiple approaches, they were assigned to the dominant methodological framework or categorized as hybrid models when integration was central to the approach. Secondary methodological features were recorded separately where studies combined multiple approaches. This taxonomy enabled systematic comparison across studies while preserving key methodological distinctions.

A narrative synthesis approach was employed to identify recurring methodological patterns, geographic disparities, data integration practices, and reported limitations across studies. Quantitative summaries (counts and proportions) were used to describe global trends.

### 2.6. Assessment of Methodological Quality

Given the methodological heterogeneity of spatio-temporal modeling studies, a formal risk-of-bias tool was not applied. Instead, we conducted a structured quality appraisal based on (i) transparency of model specification, (ii) clarity of data provenance, (iii) treatment of uncertainty, and (iv) reproducibility indicators (code or workflow availability). These criteria were used descriptively to contextualize findings rather than to exclude studies.

### 2.7. Assessment of Openness, FAIR Alignment, and Equity

To assess openness and reproducibility, we examined whether studies relied on publicly accessible data sources, documented data provenance, and provided sufficient methodological detail to support reuse. Although explicit adherence to FAIR (Findable, Accessible, Interoperable, Reusable) principles was rarely stated, and while studies were generally findable, indicators of accessibility, interoperability, and reusability were often limited, indicators such as data accessibility, use of standardized formats, availability of metadata, and reporting of code or workflows were recorded where available [12].

Equity-related considerations were assessed indirectly through geographic coverage, scale of analysis, and inclusion of socio-demographic or socioeconomic variables. Attention was paid to the representation of low- and middle-income regions and to whether studies addressed spatial inequalities in disease burden, access to testing, or healthcare resources [8,13].

### 2.8. Limitations of the Review

Several limitations should be acknowledged. First, restricting inclusion to English-language publications may have resulted in underrepresentation of studies published in other languages. Second, although multiple databases were searched, relevant studies indexed elsewhere may have been missed. Third, preprints were excluded unless subsequently peer reviewed, potentially omitting early but unvalidated contributions. Fourth, the review also did not perform quantitative meta-analysis because of substantial heterogeneity in modeling frameworks, data sources, spatial scales, and outcome definitions. Fifth, the focus on publicly available data excluded studies relying on proprietary health records, which may contain additional insights. Sixth, study selection and data extraction were not performed in duplicate, which may introduce selection bias despite the use of systematic screening criteria. Finally, a formal risk-of-bias assessment using standardized tools was not conducted due to the heterogeneity of modeling approaches and outcomes across studies. Instead, a qualitative appraisal of methodological transparency and reproducibility was applied. While appropriate for this type of methodological synthesis, this limits the ability to formally compare study quality or to weight conclusions based on risk of bias. These factors should be considered when interpreting the findings. Finally, some classification variables (e.g., geographic assignment, data streams, and methodological categorization) were inferred from study descriptions, which may introduce minor misclassification despite systematic application of standardized criteria.

The search and screening procedure is outlined in the PRISMA flow diagram (Figure 1). There were 985 records identified by database searching. After exclusion of duplicates and full-text evaluation, a total of 363 studies met the inclusion criteria. For qualitative synthesis, a purposive subsample of approximately 70 studies was examined in greater methodological depth, while quantitative summaries were based on the full corpus. This restrictiveness reflects the high methodological standard applied in the review: only those peer-reviewed articles that solely utilized spatio-temporal models and relied on publicly available COVID-19-related data (such as surveillance, mobility, environmental, or genomic data) were used. Studies that were purely temporal or purely spatial, or that relied on proprietary or inaccessible data, were excluded. These procedures ensured that the final set of studies is the most relevant and methodologically sound contributions to spatio-temporal COVID-19 modeling.

## 3. Results

### 3.1. Overview of Included Studies

**Figure 2:**
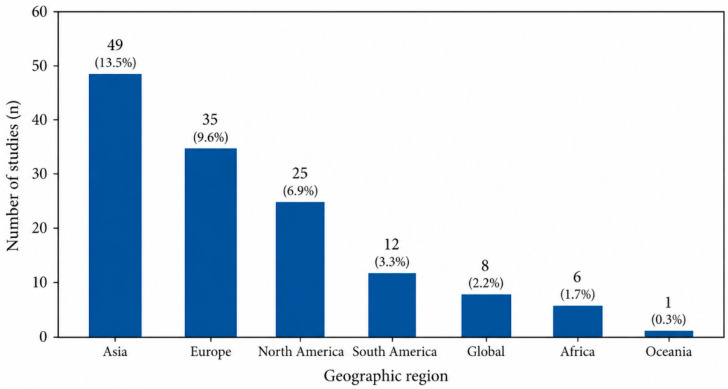
Distribution of studies by geographic region (*n* = 363). Numbers above bars indicate the number of studies and the corresponding percentage of the total (*n* = 363). Studies may include multiple regions; classification reflects the primary geographic focus reported by authors.

A total of 363 spatio-temporal COVID-19 modeling studies published between January 2020 and August 2025 met the inclusion criteria (Figure 1). These studies spanned six continents and represented substantial methodological and geographic diversity. Publication activity peaked in 2021, reflecting the urgency of early-pandemic forecasting, and declined thereafter as modeling efforts shifted toward refinement, evaluation, and retrospective synthesis (Figure 2).

The geographic distribution of studies was uneven (Figure 2). Most studies were concentrated in Asia, Europe, and North America, while relatively few focused on South America and Africa. A substantial number of studies either adopted a global perspective or did not specify a single geographic focus, reflecting the use of multi-country datasets or generalized modeling frameworks. This distribution highlights persistent underrepresentation of low- and middle-income regions.

Where studies were conducted in African contexts, they often relied on spatial econometric or panel data approaches to capture cross-country dependence in COVID-19 outcomes, as illustrated by spatial panel data modeling of infection dynamics across African countries. Countries with early and severe outbreaks, such as Italy, China, the United States, the United Kingdom, India, and Sweden, produced the largest number of studies.

Most studies were conducted at the national level, with relatively few at subnational scales. Global (*n* = 8) and multinational (*n* = 3) analyses were uncommon. When spatial scale was not explicitly reported by the authors, studies were classified as national based on the use of country-level data. For a percentage breakdown, see also Table 1.

Subnational analyses were common, including county-level studies in Sweden [14,15,16], provincial analyses in Italy [17,18,19], and city-level applications in Bangladesh and Rwanda [20,21]. Early regional mapping studies in England similarly documented strong spatial and temporal heterogeneity in COVID-19 incidence during the first pandemic wave, highlighting the value of subnational surveillance for situational awareness [22].

### 3.2. Temporal Evolution of Modeling Activity

The trajectory of spatio-temporal COVID-19 modeling followed four broad phases. Early studies in 2020 were largely exploratory, relying on classical SEIR-type compartmental models and basic spatial regressions to describe initial outbreak dynamics [1,23]. Alongside these approaches, some early studies experimented with unconventional temporal and network-based techniques to characterize epidemic spread, including spline-based and complex-network formulations applied to national case trajectories [24]. In 2021, publication volume surged, driven by short-term forecasting needs and the rapid release of case and mobility data early in the pandemic.

**Figure 3:**
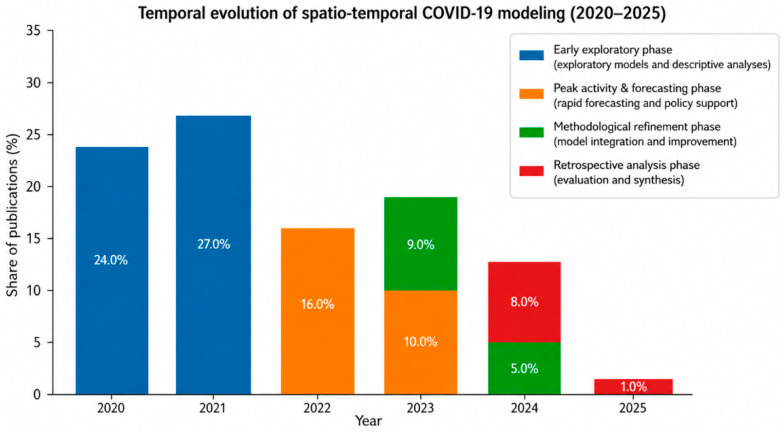
Temporal evolution of spatio-temporal modeling approaches in COVID-19 research. Percentages indicate the proportion of total reviewed studies (*n* = 363) published in each year and phase. Phases reflect the dominant focus of spatio-temporal COVID-19 modeling over time.

As shown in Figure 3, methodological diversity increased over time, with a substantial share of studies comprising conceptual, infrastructural, or non-predictive analytical contributions.

The stacked bars in Figure 3 show the number of reviewed studies by modeling family per publication year, illustrating a progression from early compartmental and Bayesian approaches toward increasing methodological diversity, including machine learning and graph-based models. The category “Other” includes conceptual contributions, data–infrastructure and surveillance system descriptions, and non-predictive analytical studies.

During 2022–2023, modeling efforts increasingly emphasized methodological refinement and data integration, incorporating vaccination coverage, viral variants, and alternative surveillance streams such as wastewater and genomics [25,26,27,28]. By 2024–2025, publication rates declined sharply as the field shifted toward retrospective evaluation, synthesis, and lessons learned.

### 3.3. Methodological Heterogeneity and Scale

The reviewed studies were classified according to the taxonomy of spatio-temporal model families defined in Section 2.5. The reviewed literature exhibited substantial methodological heterogeneity, which was strongly associated with geographic scale. SEIR/SIRD compartmental models dominated national-level analyses, particularly for policy simulation and intervention evaluation [23,29,30]. Bayesian spatio-temporal and hierarchical disease-mapping models were most applied at regional or county scales, where they supported small-area risk estimation and uncertainty quantification [14,31,32].

Alternative Bayesian formulations were also explored, including object-oriented Bayesian network models that represented spatio-temporal dependencies explicitly and supported probabilistic reasoning about COVID-19 spread at subnational scales, as demonstrated in applications to the Italian outbreak [33].

Machine learning and deep learning approaches were increasingly applied [5,34].

More recent work introduced dynamically adaptive spatio-temporal graph networks that updated spatial connectivity over time, improving robustness to changing transmission patterns and mobility structures in COVID-19 forecasting [35]. Early deep learning applications relied on long short-term memory (LSTM) networks to forecast COVID-19 incidence at the county level, capturing temporal dynamics across spatially disaggregated units in large national settings such as the United States [36].

Graph-based spatio-temporal approaches were also explored outside fully neural architectures, for example, through spectral graph wavelet methods that modeled COVID-19 dynamics across interconnected regions in the United States, capturing both spatial structure and temporal evolution [37]. At fine urban scales, Bayesian deep learning approaches were used to jointly model spatio-temporal dynamics and predictive uncertainty, enabling short-term forecasting while explicitly quantifying uncertainty in dense metropolitan settings such as Shanghai [38]. Agent-based models were typically restricted to fine spatial scales (cities, facilities), where individual-level contact patterns could be explicitly represented [39,40]. These scale–method alignments reflect trade-offs between computational complexity, data resolution, and policy relevance.

To facilitate comparison across the diverse modeling approaches identified in this review, Table 2 summarizes the main characteristics, strengths, limitations, and typical applications of major spatio-temporal model families.

### 3.4. Data Streams and Integration

Across the literature, case incidence data formed the foundation of nearly all models, regardless of method or scale [41]. Mobility data, derived from mobile phones, transportation systems, or traffic sensors, was the second most frequently used data stream and was particularly important for forecasting spatial spread and identifying inter-regional spillover [13,42,43]. At the urban scale, alternative mobility proxies such as traffic sensor data from smart city infrastructures were also integrated into spatio-temporal deep learning models, enabling fine-grained short-term forecasting without reliance on individual-level mobility traces [44].

Mortality and excess death data were primarily used in Bayesian frameworks for spatial risk mapping and severity analysis [32,45]. Hospitalization and ICU data were integrated in several studies to link transmission dynamics with healthcare system burden [14,43]. More recent graph-based deep learning models extended spatio-temporal forecasting to clinically relevant outcomes such as test positivity and hospitalization, using multi-scale graph neural networks to capture dependencies across spatial resolutions [34,35]. A smaller subset of studies incorporated genomic data to track variant emergence [28] or wastewater surveillance as an early warning signal [25,26]. Other studies explored the integration of environmental covariates, such as meteorological variables, into spatio-temporal forecasting models to improve short-term prediction accuracy at high spatial resolution [46].

Despite the availability of diverse open data sources, multi-stream integration remained limited. Several studies instead focused on identifying spatio-temporal clusters and hotspots using weighted clustering and similarity-based approaches applied to case incidence data, supporting localized risk assessment without explicit forecasting [47]. Despite the availability of diverse open data sources, the extent of data integration varied. Approximately 30% of studies relied on a single data stream, while around 70% incorporated multiple sources. However, most multi-source approaches combined only two data types, and relatively few studies integrated three or more. Comprehensive multi-source data fusion therefore remained limited, despite widespread availability of heterogeneous data.

### 3.5. Informing Public Health Interventions

A consistent finding across studies was that geographic granularity improved policy relevance. In Sweden, county-level spatio-temporal models revealed persistent spatial inequalities that were obscured by national averages [15,16]. In Italy, spatial clustering analyses demonstrated strong cross-provincial dependencies in early outbreak dynamics [17,47]. Bayesian disease-mapping in Peru and Bangladesh identified high-risk regions and vulnerable populations despite limited data environments [20,45].

Mobility-informed models frequently detected emerging hotspots earlier than models based solely on case counts and were used to guide targeted interventions, including travel restrictions and localized lockdowns [13,42,48]. More broadly, data-driven spatio-temporal forecasting frameworks were developed explicitly to support government policy making by providing short-term projections under different intervention scenarios [49,50]. Comparative counterfactual analyses further demonstrated the utility of spatio-temporal modeling for evaluating policy choices, such as contrasting national lockdown strategies across Sweden, Denmark, and the UK [51].

### 3.6. Limits of Predictive Performance

Although spatio-temporal models were effective for short-term forecasting, many struggled with abrupt structural changes. The emergence of new variants, most notably Omicron, exposed limitations in models calibrated under earlier conditions [1]. Even sophisticated machine learning and mechanistic models exhibited reduced accuracy beyond a few weeks, particularly when behavioral patterns or policy regimes shifted rapidly [1,52]. In addition, medium-term changes in internal population movements during the pandemic altered baseline mobility structures in several Latin American countries, reducing the validity of pre-pandemic mobility assumptions embedded in many spatio-temporal models [53]. These findings underscore that spatio-temporal models are most reliable for near-term situational awareness and scenario exploration, rather than long-range prediction under high uncertainty.

### 3.7. Socio-Demographic Factors in Spatio-Temporal COVID-19 Models

Many studies demonstrated that incorporating socio-demographic and socioeconomic variables substantially improved model performance and interpretability. Population density, housing conditions, income, testing access, and occupational exposure were consistently associated with spatial variation in infection and mortality rates [15,16]. Syndemic-oriented analyses highlighted how COVID-19 amplified existing social vulnerabilities, particularly in urban and marginalized communities [8]. In Latin America, mobility reductions and infection rates were strongly stratified by socioeconomic status, limiting the effectiveness of uniform interventions [42,54]. These findings reinforce the importance of embedding social context within spatio-temporal modeling frameworks.

In addition to conventional population density, several studies have highlighted the importance of how population is spatially distributed within administrative units. Population-weighted density (PWD), which reflects the density experienced by the average individual, provides a more realistic proxy for potential contact intensity than aggregate density measures. This distinction is particularly relevant in spatio-temporal COVID-19 modeling, where transmission dynamics depend on localized interaction structures rather than average population distribution. Recent work shows that PWD is more strongly associated with COVID-19 infection rates than traditional density metrics, suggesting that it may improve the interpretation of spatial heterogeneity in transmission [55]. In practice, failure to account for this distinction may lead to underestimation of transmission risk in highly concentrated urban areas and overestimation in sparsely populated regions. Within the studies reviewed here, population density was frequently included as a covariate, but rarely operationalized in ways that capture intra-regional variation, indicating an important limitation in how spatial structure is represented in many spatio-temporal models.

### 3.8. Openness, FAIR Practices, and Collaboration

Nearly all reviewed studies relied on publicly available data, yet explicit adherence to FAIR principles was uncommon, see Table 3 [12,56]. While data sources were generally identifiable, accessibility, interoperability, and reusability were often limited.

Adherence to FAIR principles (Findable, Accessible, Interoperable, Reusable) and ethical considerations was assessed qualitatively using predefined operational criteria (Table 3). Each study was categorized as having high, moderate, or low adherence for each principle based on the clarity of data sourcing, accessibility conditions, use of standardized formats, and availability of reproducible workflows. Ethical considerations were assessed based on whether studies explicitly or implicitly addressed issues such as privacy, bias, or policy implications. The assessment was conducted descriptively by a single reviewer and used to identify general patterns across the literature rather than to provide a formal quantitative benchmark. Nevertheless, the pandemic fostered unprecedented open collaboration. Cross-national data sharing, open-source code release, and shared dashboards enabled rapid methodological diffusion and comparative evaluation [56]. Operational examples of automated spatio-temporal surveillance systems further demonstrate how modeling outputs can be translated into routine public health action, as illustrated by Denmark’s national COVID-19 surveillance infrastructure integrating continuous data flows for situational awareness and decision support [57]. Novel data sources such as social media signals and call-center data were explored to supplement traditional surveillance in data-scarce settings, albeit with ethical and representativeness challenges.

## 4. Discussion

These findings underscore that spatio-temporal models help quantify spatial heterogeneity in risk, healthcare access, and vulnerability, enabling structured analysis of geographically patterned disparities. The COVID-19 pandemic accelerated the operational adoption of spatio-temporal modeling in public health decision-making. Across diverse geographic contexts, the studies reviewed here demonstrate that explicitly integrating space and time improved situational awareness, supported targeted interventions, and revealed spatially structured disparities in infection risk and healthcare access that would have remained obscured in non-spatial or purely temporal analyses. At the same time, the literature exposes persistent methodological and infrastructural challenges that limit the long-term utility and transferability of these approaches.

### 4.1. Open Data, FAIR Principles, and Cross-Border Comparability

A central lesson from the pandemic concerns the role of open and FAIR-aligned data infrastructures. Publicly accessible case counts, mobility reports, genomic databases, and wastewater surveillance enabled rapid development and deployment of spatio-temporal models across many settings [25,42,56]. However, while data accessibility was generally high, interoperability and reusability were frequently constrained by inconsistent metadata standards, shifting case definitions, unstable geographic boundaries, and limited documentation of workflows [12,56]. As a result, cross-national comparisons and model reuse were often difficult, even when data were nominally open.

Initiatives such as the European Open Science Cloud (EOSC) demonstrated how harmonized, machine-readable infrastructures can support cross-border modeling and rapid reuse, but these efforts remain unevenly implemented and concentrated in high-income regions. The findings of this review suggest that FAIR principles must extend beyond datasets alone to encompass models, workflows, and documentation if spatio-temporal modeling is to become a standing capacity rather than an ad hoc crisis response. Embedding FAIR compliance into routine public health data infrastructures would improve reproducibility, facilitate transferability, and reduce duplication of effort during future emergencies.

Importantly, FAIR alignment also intersects with privacy, data governance, and representativeness considerations. Several studies highlighted the need for privacy-preserving approaches, such as aggregated mobility indicators or federated learning, to balance analytical power with individual rights [56,58]. Inclusion of socio-demographic variables improves representativeness and reduces geographic bias in model inference.

### 4.2. Scale, Spatial Units, and the Limits of Administrative Geography

Geographic scale emerged as a defining factor in both model performance and policy relevance. National-level analyses were effective for identifying broad epidemic waves and informing high-level policy, but frequently masked substantial local heterogeneity. In contrast, regional and neighborhood-scale models revealed persistent spatial inequalities in infection risk, mortality, and access to testing or care [15,16,22]. These findings reinforce long-standing insights from health geography that epidemics are experienced locally, even when managed nationally. These challenges are further compounded by spatial misalignment between datasets (e.g., administrative boundaries, mobility units, and health reporting units), which complicates integration and may introduce bias in spatio-temporal inference.

At the same time, heavy reliance on administratively defined spatial units exposed analyses to the Modifiable Areal Unit Problem (MAUP), whereby results vary depending on the choice of spatial aggregation [59]. Because administrative boundaries rarely align with patterns of human mobility or social interaction, models constrained to these units may obscure cross-border transmission and spillover effects. Studies that incorporated mobility flows, network structures, or continuous spatial surfaces often provided more realistic representations of epidemic diffusion, highlighting the importance of testing sensitivity to scale as a standard modeling practice.

These results suggest that future spatio-temporal models should treat spatial units as analytical choices rather than fixed entities, explicitly evaluating how scale and boundary definitions influence inference and policy recommendations.

### 4.3. Forecasting, Uncertainty, and Model Humility

While spatio-temporal models proved effective for short-term forecasting and situational awareness, their limitations became evident during periods of rapid structural change. The emergence of new variants, most notably Omicron, exposed the fragility of models calibrated under earlier epidemiological regimes [1,52]. Even sophisticated mechanistic and machine learning frameworks struggled to anticipate abrupt shifts driven by viral evolution, behavioral change, or policy interventions. Only a minority of reviewed studies reported formal validation metrics such as cross-validation, calibration assessment, or posterior predictive checks, limiting comparability of predictive performance across modeling families.

These failures should not be interpreted as shortcomings of individual models but as structural features of epidemic systems characterized by non-stationarity and deep uncertainty. To address this challenge, probabilistic spatio-temporal neural networks were developed to produce full predictive distributions rather than point estimates, enabling uncertainty-aware short-term forecasting of COVID-19 counts [60]. The findings of this review support a shift away from long-range point prediction toward probabilistic forecasting, scenario analysis, and transparent communication of uncertainty. In this context, the value of spatio-temporal modeling lies less in precise prediction than in supporting adaptive decision-making under uncertainty.

### 4.4. Social Determinants and Spatial Inequality

Across regions, models that incorporated socio-demographic and socioeconomic variables consistently produced more nuanced and policy-relevant insights. Population density, housing conditions, occupational exposure, income, and testing accessibility were strongly associated with spatial variation in COVID-19 incidence and mortality [15,16]. Syndemic-oriented analyses further demonstrated how COVID-19 interacted with pre-existing social vulnerabilities, amplifying inequalities within and between cities [8].

These findings demonstrate that incorporating socio-demographic structure improves explanatory power and policy relevance in spatio-temporal models rather than purely biological phenomena. Spatio-temporal models that ignore social context risk reproducing structural biases, while those that integrate social determinants are better positioned to inform equitable interventions and resource allocation.

### 4.5. Collaboration and Open Science

Finally, the pandemic demonstrated the power of open collaboration. Rapid sharing of data, code, and modeling outputs enabled unprecedented methodological diffusion and comparative analysis across regions [56]. Global platforms for genomic surveillance, mobility reporting, and open dashboards illustrated how collective infrastructure can accelerate scientific response. At the same time, persistent digital divides limited participation from low- and middle-income regions, reinforcing existing inequalities in modeling capacity [8]. Sustained investment in open, interoperable, and inclusive infrastructures is therefore essential if spatio-temporal modeling is to support global preparedness rather than exacerbate disparities.

### 4.6. Implications for Global Health Practice

Effective epidemic decision-support requires scale-appropriate modeling, probabilistic forecasting under uncertainty, interoperable multi-source data integration, and embedding outputs within GIS-based operational platforms.

Pandemic preparedness depends on interoperable data infrastructures that are findable, accessible, interoperable, and reusable, while incorporating privacy and representativeness safeguards. Long-term forecasting should be used with caution. Spatio-temporal models are most reliable for short-term situational awareness and scenario exploration; long-range predictions should be probabilistic and accompanied by transparent communication of uncertainty.

Uneven surveillance capacity and data availability constrain model generalizability and cross-context transferability. Limited surveillance capacity and uneven data availability in low- and middle-income regions constrain both model performance and policy relevance unless actively addressed.

Multi-source data integration improves spatial sensitivity. Combining epidemiological data with mobility, genomic, or wastewater surveillance enhances early detection and supports adaptive responses during periods of rapid epidemiological change.

Embedding models in GIS-based decision-support systems increases impact. Translating spatio-temporal analyses into place-based, accessible tools facilitates uptake by public health practitioners and supports geographically targeted action.

## 5. Conclusions

This review synthesizes spatio-temporal approaches used to analyze COVID-19 dynamics across scales, data types, and modeling paradigms. By examining five years of global spatio-temporal COVID-19 modeling, it contributes to ongoing methodological debates regarding scale, spatial dependence, and decision-support integration. The findings demonstrate that spatio-temporal modeling became a cornerstone of pandemic analysis, accelerating the integration of spatial epidemiology into public health practice. Across 363 studies published between 2020 and 2025, models integrating space and time—ranging from Bayesian disease mapping and SEIR-type compartmental frameworks to machine learning, graph-based, and agent-based approaches—played a central role in understanding epidemic diffusion, identifying hotspots, evaluating interventions, and revealing persistent socio-spatial inequalities.

A central finding of this review is that geography mattered at every stage of the pandemic, but not uniformly. Models operating at finer spatial scales consistently generated more actionable insights than national-level analyses, particularly when they incorporated human mobility, demographic context, and local health system capacity. At the same time, heavy reliance on administratively defined spatial units exposed analyses to scale-related distortions, underscoring the need to explicitly address the Modifiable Areal Unit Problem (MAUP) and to test sensitivity across spatial resolutions [15,29]. These findings reaffirm that epidemics unfold through networks of places rather than within fixed administrative boundaries.

The review also highlights the pivotal role of open data in enabling rapid modeling during the pandemic. Publicly available case counts, mobility datasets, genomic repositories, and wastewater surveillance made spatio-temporal analysis possible across diverse contexts. However, despite widespread use of open data, adherence to FAIR principles (Findable, Accessible, Interoperable, Reusable) was uneven. While data sources were generally findable, accessibility, interoperability, and reusability were frequently limited by inconsistent metadata, shifting definitions, and insufficient documentation of workflows. These constraints hindered cross-border comparability and model transferability, indicating that openness alone is insufficient without coordinated data standards and reproducible infrastructures [10,55].

Importantly, the pandemic exposed substantial geographic disparities in both data availability and modeling activity. Studies were concentrated in high-income regions, with comparatively limited representation of low- and middle-income contexts. This imbalance reflects structural inequalities in data infrastructure rather than differences in methodological capacity. Where models incorporated socio-demographic variables, they consistently showed that COVID-19 outcomes were shaped by underlying inequalities in population structure, mobility, and access to healthcare and testing [8,59]. These findings emphasize that spatio-temporal models must be interpreted within their social and data contexts, as model outputs may partially reflect surveillance gaps and uneven data quality.

The review further shows that data integration was more common than often assumed but remained limited in depth. While most studies combined multiple data sources, most integrations involved only two data streams, and comprehensive multi-source fusion was relatively rare. This suggests that the potential of heterogeneous data integration was only partially realized during the pandemic. Future work should prioritize more systematic integration of complementary data streams, including mobility, genomic, environmental, and socio-demographic data.

Forecasting performance revealed important limitations of predictive modeling in rapidly evolving systems. While many models performed well for short-term projections, most struggled to anticipate abrupt structural changes, such as the emergence of new variants or sudden behavioral shifts [1,51]. These findings highlight inherent limits of predictive modeling in non-stationary epidemic systems: spatio-temporal models are best used to support adaptive decision-making, scenario exploration, and uncertainty-aware planning rather than long-term deterministic prediction.

Taken together, the findings of this review suggest that future pandemic preparedness depends on embedding spatio-temporal modeling within FAIR-aligned data infrastructures that are not only findable, but also accessible, interoperable, reusable, and ethically grounded. This includes investing in harmonized cross-border data standards, improving reproducibility through shared workflows and code, integrating multiple surveillance streams, and systematically incorporating social determinants of health. Geographic Information Systems (GIS), when coupled with spatio-temporal models, provide a critical decision-support framework by translating complex analyses into actionable, place-based intelligence.

In conclusion, COVID-19 demonstrated that epidemic dynamics are intrinsically spatio-temporal and sensitive to geographic scale, data structure, and model specification. Models that explicitly represent spatial dependence, temporal evolution, and socio-demographic heterogeneity provide more reliable situational awareness than approaches that ignore these components. Strengthening reproducibility, uncertainty quantification, multi-source integration, and cross-context transferability will be central to advancing spatio-temporal epidemic modeling in future global health emergencies.

## Figures and Tables

**Table 1:** Spatial scales of articles.

Spatial Scale	Number of Studies (*n*)	Percentage (%)
National	315	86.8
City (local)	17	4.7
Regional (subnational)	15	4.1
Global	8	2.2
Multinational	3	0.8
Total	363	100.0

**Table 2:** Comparison of spatio-temporal COVID-19 modeling approaches based on the taxonomy of model families defined in Section 2.5.

Model Family	Key Characteristics	Strengths	Typical Applications
Bayesian/statistical spatio-temporal models	Explicit modeling of spatial dependence and temporal dynamics (e.g., hierarchical, regression-based, disease-mapping approaches)	Strong uncertainty quantification; high interpretability; suitable for small-area analysis	Risk mapping; surveillance; hotspot detection; small-area estimation
Compartmental models (SEIR/SIRD variants)	Mechanistic epidemic models representing population transitions between disease states	Interpretable epidemic dynamics; useful for scenario analysis and policy simulation	Intervention assessment; epidemic trajectory simulation; scenario modeling
Machine learning/deep learning models	Data-driven nonlinear models (e.g., LSTM, CNN, transformers, ensemble methods) without explicit epidemiological structure	Strong short-term predictive performance; flexible with large, complex datasets	Short-term forecasting; pattern recognition; early warning systems
Graph-based spatio-temporal models	Network-based representations of spatial structure (e.g., graph neural networks, mobility networks) capturing inter-regional connectivity	Captures spatial interactions and connectivity; suitable for modeling spread across networks	Spread analysis; mobility-informed forecasting; network diffusion modeling
Agent-based models	Individual-level simulations of interactions, movement, and transmission processes	High realism; captures heterogeneity and local dynamics	Local intervention testing; facility- or city-level simulations; policy scenario testing
Mechanistic–statistical hybrid models	Integration of mechanistic epidemic structure with statistical or data-driven components	Balances interpretability and predictive flexibility; improves robustness	Hybrid forecasting; data assimilation; intervention evaluation

**Table 3:** The FAIR + E principles.

Principle	Operational Definition (in This Review)	High Adherence	Moderate Adherence	Low Adherence	Key Observations
Findable (F)	Data sources clearly identifiable and traceable (e.g., named datasets, DOIs, repositories)	99%	1%	0%	Nearly all studies include DOIs or clearly reference identifiable datasets, making data sources highly traceable across the literature.
Accessible (A)	Data publicly available or accessible without major restrictions	1%	4%	95%	Despite reliance on public data, explicit reporting of accessibility is rare; most studies do not clearly specify access conditions or provide direct links to datasets.
Interoperable (I)	Use of standardized formats, harmonized variables, or integration across datasets	12%	28%	60%	Interoperability remains limited; many studies rely on heterogeneous data structures and lack standardized frameworks for integration.
Reusable (R)	Availability of code, detailed methods, or reproducible workflows	2%	25%	73%	Reproducibility is a major limitation; few studies provide code or workflows sufficient for full reuse or replication.
Ethical (+E)	Explicit or implicit consideration of ethics (privacy, bias, policy implications)	18%	42%	40%	Ethical considerations are often implicit rather than explicitly addressed, with limited discussion of bias, privacy, or data governance.

## Data Availability

All data used in this study are derived from published literature included in the systematic review. The dataset generated during screening and extraction is available from the corresponding author upon reasonable request.

## Supplementary Materials

The following supporting information can be downloaded at: https://www.mdpi.com/article/10.3390/ijerph23050627/s1, File S1: PRISMA 2020 Checklist. File S2: Complete dataset of included studies and extracted study characteristics. File S3: Additional references for studies included in the review corpus but not directly discussed in the main text. Additional studies included in the review corpus are cited here [61,62,63,64,65,66,67,68,69,70,71,72,73,74,75,76,77,78,79,80,81,82,83,84,85,86,87,88,89,90,91,92,93,94,95,96,97,98,99,100,101,102,103,104,105,106,107,108,109,110,111,112,113,114,115,116,117,118,119,120,121,122,123,124,125,126,127,128,129,130,131,132,133,134,135,136,137,138,139,140,141,142,143,144,145,146,147,148,149,150,151,152,153,154,155,156,157,158,159,160,161,162,163,164,165,166,167,168,169,170,171,172,173,174,175,176,177,178,179,180,181,182,183,184,185,186,187,188,189,190,191,192,193,194,195,196,197,198,199,200,201,202,203,204,205,206,207,208,209,210,211,212,213,214,215,216,217,218,219,220,221,222,223,224,225,226,227,228,229,230,231,232,233,234,235,236,237,238,239,240,241,242,243,244,245,246,247,248,249,250,251,252,253,254,255,256,257,258,259,260,261,262,263,264,265,266,267,268,269,270,271,272,273,274,275,276,277,278,279,280,281,282,283,284,285,286,287,288,289,290,291,292,293,294,295,296,297,298,299,300,301,302,303,304,305,306,307,308,309,310,311,312,313,314,315,316,317,318,319,320,321,322,323,324,325,326,327,328,329,330,331,332,333,334,335,336,337,338,339,340,341,342,343,344,345,346,347,348,349,350,351,352,353,354,355,356,357,358,359,360,361,362,363,364,365,366,367,368,369,370,371,372,373].

## Abbreviations

The following abbreviations are used in this manuscript:

COVID-19	Coronavirus Disease 2019
GIS	Geographic Information Systems
SEIR	Susceptible–Exposed–Infectious–Recovered
SIRD	Susceptible–Infectious–Recovered–Deceased
FAIR	Findable, Accessible, Interoperable, Reusable
PRISMA	Preferred Reporting Items for Systematic Reviews and Meta-Analyses
CNN	Convolutional Neural Network
LSTM	Long Short-Term Memory
ICU	Intensive Care Unit
MAUP	Modifiable Areal Unit Problem
