# Supplementary material for: Spatio-Temporal COVID-19 Modeling: A Global Systematic Review of Data Integration, Equity, and Lessons for Pandemic Preparedness
Source: Int J Environ Res Public Health. 2026 May 8;23(5):627. doi: 10.3390/ijerph23050627 (PMC13205768; doi:10.3390/ijerph23050627)
Supplement: Supplementary file 1 [file ijerph-23-00627-s001.zip › File S1 PRISMA 2020 Checklist.pdf]

PRISMA 2020 Checklist (Simplified)

Section	PRISMA Item	Reported	Page(s) in Manuscript
Title	Identify the report as a systematic review	Yes	**Page 1**
Abstract	Structured summary	Yes	**Page 1**
Introduction – Rationale	Describe rationale	Yes	**Page 3**
Introduction – Objectives	State objectives	Yes	**Page 3–4**
Methods – Eligibility criteria	Inclusion/exclusion criteria	Yes	**Page 6–7**
Methods – Information sources	Databases and sources	Yes	**Page 6**
Methods – Search strategy	Full search strategy	Yes	**Page 6**
Methods – Selection process	Screening procedure	Yes	**Page 7**
Methods – Data collection	Data extraction	Yes	**Page 7–8**
Methods – Study risk of bias	Quality appraisal	Yes	**Page 8**
Methods – Synthesis methods	Synthesis approach	Yes	**Page 7–8**
Results – Study selection	Numbers screened/included	Yes	**Page 12**
Results – Study characteristics	Description of studies	Yes	**Page 12–14**
Discussion – Limitations	Limitations of review	Yes	**Page 8 (Methods 2.8)** and **Page 19–20**

Discussion – Conclusions	Interpretation/implications	Yes	**Page 24**
Other – Funding	Funding sources	Yes	**Page 28**
